# Roll-to-Roll Manufacturing of Micropatterned Adhesives by Template Compression

**DOI:** 10.3390/ma12010097

**Published:** 2018-12-29

**Authors:** Dan Yu, Dirk Beckelmann, Michael Opsölder, Bruno Schäfer, Karsten Moh, René Hensel, Peter William de Oliveira, Eduard Arzt

**Affiliations:** 1 INM—Leibniz Institute for New Materials, Campus D2 2, 66123 Saarbrücken, Germany; dan.yu@leibniz-inm.de (D.Y.); dirk.beckelmann@leibniz-inm.de (D.B.); michael.opsoelder@leibniz-inm.de (M.O.); bruno.schaefer@leibniz-inm.de (B.S.); karsten.moh@leibniz-inm.de (K.M.); rene.hensel@leibniz-inm.de (R.H.); peter.oliveira@leibniz-inm.de (P.W.d.O.); 2 Department of Materials Science and Engineering, Saarland University, 66123 Saarbrücken, Germany

**Keywords:** dry adhesive, microstructure, roll-to-roll fabrication, biomimetics

## Abstract

For the next generation of handling systems, reversible adhesion enabled by micropatterned dry adhesives exhibits high potential. The versatility of polymeric micropatterns in handling objects made from various materials has been demonstrated by several groups. However, specimens reported in most studies have been restricted to the laboratory scale. Upscaling the size and quantity of micropatterned adhesives is the next step to enable successful technology transfer. Towards this aim, we introduce a continuous roll-to-roll replication process for fabrication of high-performance, mushroom-shaped micropatterned dry adhesives. The micropatterns were made from UV-curable polyurethane acrylates. To ensure the integrity of the complex structure during the fabrication process, flexible templates were used. The compression between the template and the wet prepolymer coating was investigated to optimize replication results without structural failures, and hence, to improve adhesion. As a result, we obtained micropatterned adhesive tapes, 10 cm in width and several meters in length, with adhesion strength about 250 kPa to glass, suitable for a wide range of applications.

## 1. Introduction

Micropatterned surfaces have attracted considerable attention as dry adhesives because of their remarkable adhesion performance. Inspired by the fibrillar attachment system of insects and geckos, the underlying mechanism of adhesion was comprehensively discussed and the governing principle of “contact splitting” was validated in numerous systems of artificial micropatterned surfaces [1,2,3]. For the rational design of synthetic micropatterned surfaces, several aspects must be considered, such as pillar dimension and tip geometry, bulk material property, backing layer thickness as well as the rigidity of the target substrate [4,5,6,7,8]. Particularly, the terminal tip of micropillars forming contact with the substrate is essential for high adhesion. In several studies, mushroom-shaped tips have been found to outperform other geometries by significantly increasing pull-off stress and work of separation [4,5,9,10,11,12,13,14,15]. The adhesion is mainly improved due to the beneficial distribution of interfacial normal stresses. In particular, the magnitude of stress singularities at the corner of the adhesive contact can be reduced in the mushroom tip design compared to non-optimized flat punch structures [16,17,18,19].

For the demonstration of practical applicability, the transfer from laboratory (~cm^2^) to large-scale (~m^2^) fabrication of mushroom-shaped microstructures is crucial [3]. This step remains challenging despite continuous progress in large area and continuous fabrication methods over the last decades [20,21,22,23,24]. Often the material selection is limited by the curing conditions in a continuous line compared to batch fabrication. For micropatterned dry adhesives, the majority of materials used in previous studies were polydimethylsiloxanes (PDMS) and polyurethanes (PUs), both of which cure by thermal crosslinking. The reason for this material choice is their suitable mechanical properties such as low elastic modulus and high elongation at break, which impart good adhesion strength combined with high flexibility [4,9,10,11,12,13,14,15]. However, considering a continuous roll-to-roll fabrication, materials of choice must allow fast curing. For this reason, ultraviolet (UV)-curable resins are promising candidates, whose curing is much faster (seconds up to few minutes) than in thermally cured systems (minutes up to few hours) [4,9,10,11,13,15,25,26,27,28]. For example, the group Kwak et al. [29,30] reported on a continuous roll-to-roll fabrication of micropatterned dry adhesives made from modulated poly(urethane acrylate) (PUA) resin [29,30]. In addition to the fast curing material, the authors used a flexible template to replicate mushroom-shaped microstructures. The utilization of flexible templates can minimize the demolding failure for imprinting the microstructures with complex geometry including reentrant features, especially for replicating high modulus materials. Such a template, however, can be sensitive to deformations induced by compressive loads during imprinting, which in turn can result in variations of the micropattern morphology and of adhesive properties. A detailed study on the deformation of flexible templates has not been reported so far. Towards this aim, we systematically vary the distance between the imprinting roll and the pressure roll, which in turn influences the pressure between the micropatterned flexible template and the wet prepolymer coating. We will report variations of the morphology in relation to the distance and correlate those with the pull-off strength obtained from adhesion measurements. 

## 2. Methods 

### 2.1. Material Preparation and Characterization

Aliphatic urethane diacrylate oligomer, Miramer UA5216 (UA16, Miwon Specialty Chemical Co. Ltd., Gwanggyo, South Korea), was mixed with 5 wt % photoinitiator, Omnirad 500 (IGM Resins B.V., Waalwijk, The Netherlands) under vacuum for 3 min and used as prepolymer for the micropatterned dry adhesives. The two-component polydimethylsiloxane Elastosil^®^ M4601 (ePDMS, Wacker Chemie AG, München, Germany) was mixed before usage under vacuum for 3 min and used for the flexible templates. The ePDMS prepolymer was thermally cured at 70 °C for 1 h.

For dynamic mechanical thermal analysis (DMTA) measurements (Q800, TA Instruments, New Castle, DE, United States of America), three samples with the dimension of 30 mm × 5.5 mm × 2 mm were prepared and storage modulus was recorded. The specimens of UA16 and ePDMS were tested in tensile mode at an oscillatory frequency of 1 Hz in the temperature range from −100 to 120 °C under N_2_ atmosphere. The elongation at break for both materials was examined by tensile tests (Zwick Roell GmbH & Co. KG, Ulm, Germany) with a crosshead moving speed of 20 mm min^−1^ until specimen fracture. For the tests, five specimens with dog bone shape (thickness: 3mm; gage length: 60 mm; gage width: 8 mm; grip width: 17 mm) were used.

The surface free energy of UA16 and ePDMS was determined by contact angle goniometry (OCA35, analysis software: SCA20, DataPhysics Instruments GmbH, Filderstadt, Germany). Specimens with smooth surfaces were prepared as follows: 120-µm thick films of UA16 prepolymer were coated onto smooth polyethylene terephthalate (PET) foil and subsequently cured for 5 min with UV light; ePDMS was cured at 70 °C for 1 h. Water and n-hexadecane (2 µl) were used as testing liquids for static contact angle measurements. Between six and ten positions on each specimen were measured. The surface free energy was deduced from the obtained contact angles by using Wu’s harmonic mean method [31]. Details of the contact angles obtained, and the calculated surface free energies are summarized in Table S1 in the Supplementary Materials.

The dynamic viscosity of the UA16 prepolymer (without blending with Omnirad 500) was measured by rheometry (Physica MCR300) in the temperature range between 25 and 80 °C with a shear rate of 1 s^−1^.

For scanning electron microscope (SEM) imaging, samples were sputter-coated with gold layers at 30 mA for 40 s using a Jeol JFC-1300 auto fine coater (Nihon Denshi K.K. / Jeol Ltd., Akishima, Japan). Secondary electron images were acquired at 5 kV accelerating voltage in high vacuum conditions using a FEI Quanta 400 FEG (image size 1024 × 884 pixels, dwell time 10 µs).

### 2.2. Flexible Template Preparation

The ePDMS prepolymer was cast into a customized circular fixture (diameter: 130 mm and depth: 5 mm), in which the master mold (Ni shim, from Temicon GmbH, Dortmund, Germany) was mounted. The ePDMS was cured at 70 °C for 1 h and, subsequently, gently demolded from the Ni shim. Several ePDMS templates were prepared by repeating this procedure and then trimmed into square shape, before they were glued onto the imprinting roll. The flexible ePDMS templates were used for the roll-to-roll fabrication of UA16 microstructures without any further surface modification.

### 2.3. Micropattern Fabrication by a Roll-to-Roll Process

**Figure 1:**
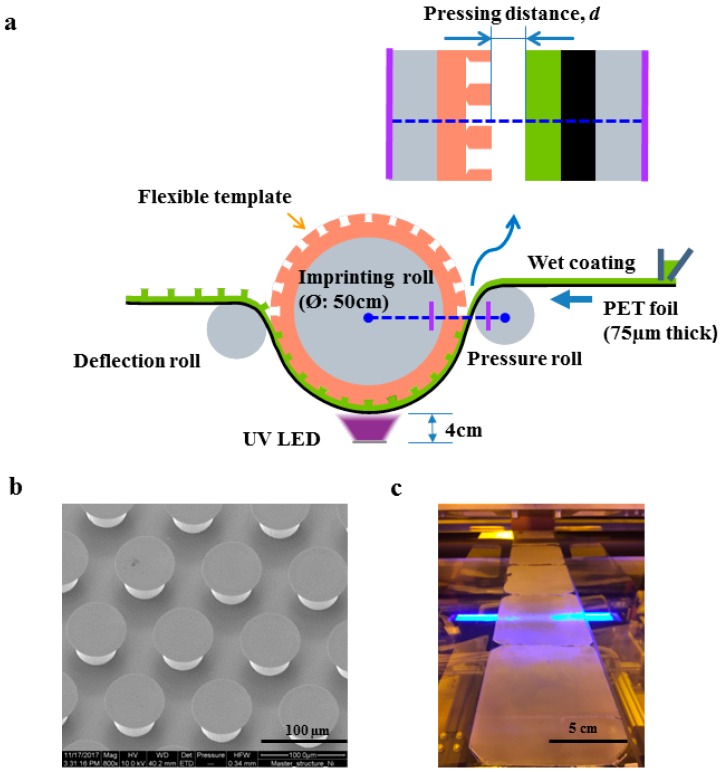
Roll-to-roll fabrication of dry adhesive films. (**a**) Illustration of the imprint unit: The flexible template was glued to the imprinting roll. The wet coating (UA16 prepolymer) on the polyethylene terephthalate (PET) film was pressed into the micropatterned template by means of the pressure roll. The surface-to-surface distance between the wet coating and the template defines the pressing distance, d. The position of the deflection roll defines the demolding angle. (**b**) Scanning electron micrographs of the Ni shim surface, providing a positive master structure for template fabrication. (**c**) Photograph of the dry adhesive film made from UA16 upon demolding.

The roll-to-roll system (Jakob Weiß & Söhne, Sinsheim, Germany) employed in this work features a total web length of 24 m from pay-off to take-up position. The pilot line consists of several functional units such as a wet coating station, a thermal drying oven, and an imprint unit, which is the key component. The custom-made imprint unit consists of the imprinting roll in between two backside rolls—a pressure roll pressing the PET film (as a substrate) with the wet prepolymer coating to the flexible template and a deflection roll guiding the delamination upon curing (Figure 1a). The 5-mm thick ePDMS templates were glued on the imprinting roll, which had a diameter of 500 mm and a width of 600 mm. The Ni shim for the replication process contained a hexagonally arranged array of mushroom-shaped micropillars with diameters and heights of 50 µm and a center-to-center distance of 100 µm (Figure 1b). The tip diameter of the mushrooms ranged from 66 to 76 µm throughout the entire array. For in-line curing, two UV-LED radiators with 600 mm length and 33 mm width, having their peak intensity of 6 W cm^−2^ at 365 nm, were placed between the two backside rolls. For the micropatterned adhesives, a PET film was used as a substrate, on which the backing layer was coated. The thickness of the UA16 prepolymer film (i.e., the wet coating) was 90 µm, set by a doctor’s blade. The web speed was 0.1 m min^−1^, providing sufficient time for UV curing.

**Figure 2:**
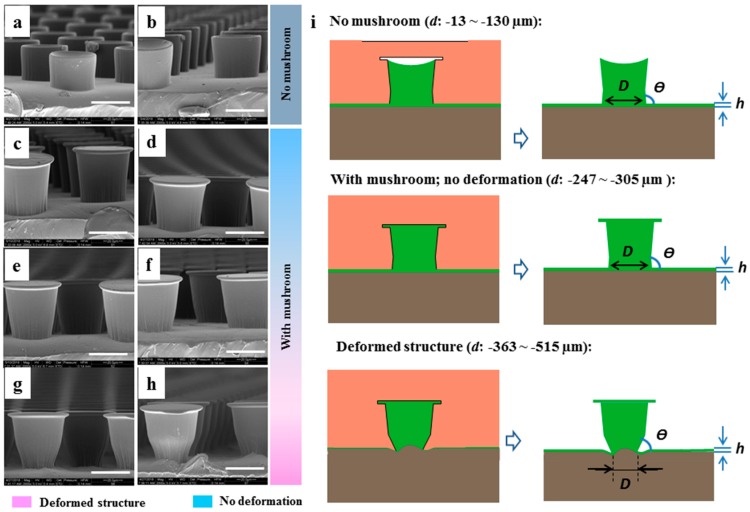
Morphology of the microstructures. Scanning electron micrographs of UA16 micropillars fabricated with different distances, *d*, between template and wet coating: (**a**) −13 µm, (**b**) −130 µm, (**c**) −247 µm, (**d**) −305 µm, (**e**) −363 µm, (**f**) −422 µm, (**g**) −468 µm, and (**h**) −515 µm. Scale bars are 40 µm. (**i**) Illustration of micropillar shape distortions due to deformation of the flexible mold during imprinting and curing (left) and upon demolding (right). These distortions led to variation of the pillar diameter at the connection to the backing layer, D, the angle between pillar sidewall and backing layer, θ, and the backing layer thickness, *h* (see Table 2).

**Figure 3:**
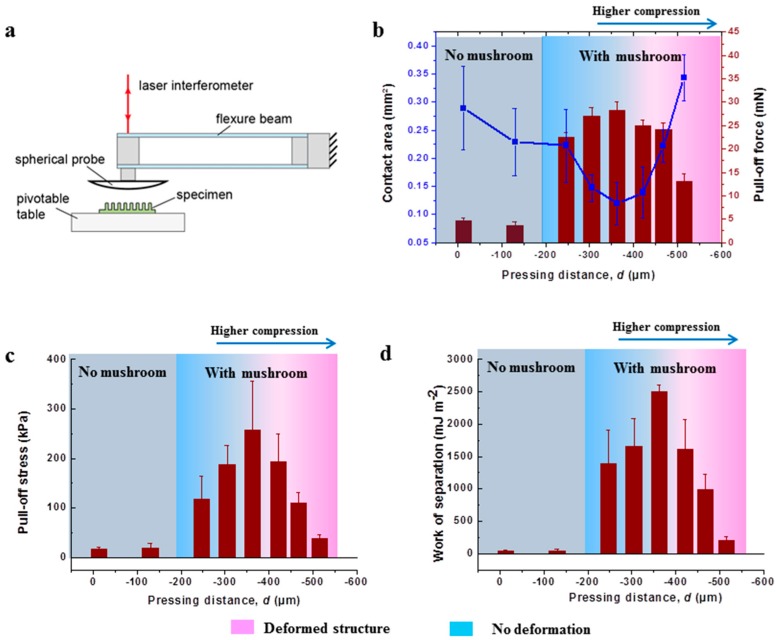
Adhesion test setup and results. (**a**) Illustration of the adhesion test device. (**b**) The pull-off force (red bars) and the contact area (blue squares) in terms of the distance between template and wet coating during roll-to-roll fabrication; the measured pull-off stress and work of separation are shown in (**c**) and (**d**), respectively. Error bars correspond to the standard deviation of the adhesion test.

Contact formation between the wet prepolymer coating and the ePDMS template for the imprint step was controlled by positioning the pressure roll with respect to the imprinting roll using a stepping motor. The surface-to-surface distance between the coating and the template was varied between +10 µm and −515 µm. The distance of 0 µm represents intimate contact of the wet coating to the template; positive and negative values represent non-contact and compressive contact, respectively. As the degree of compressive contact was essential for the quality of the microstructures, this parameter—denoted “compression”—will play a central role in the assessment of the results below. The UA16 prepolymer was cured in contact with the template by UV exposure through the PET-foil from the back side. The cured microstructures were demolded from the ePDMS template, which was controlled by the position of the deflection roll. The entire process was carried out in a UV light protected clean room facility. The obtained microstructures were examined by scanning electron micrographs for morphology inspection (Figure 2) and adhesion measurements for adhesive performances (Figure 3).

### 2.4. Adhesion Measurement

To test the adhesion, the micropatterned film was cut into pieces of 15 mm × 15 mm and glued to a glass slide with a UV adhesive (BO MV76002, Bohle AG, Haan, Germany). Test of normal adhesion were performed in a humidity and temperature-controlled laboratory (humidity: 50 ± 10%; temperature: 21 °C), using a custom-built device, which consists of an interferometer, a pivotable stage and a spherical, smooth glass probe (curvature radius: 15 mm) mounted on a glass beam (spring constant: 2241 N m^−1^). The resolution of the force sensor is about 100 µN. The illustration of the setup is shown in Figure 3a below. The compressive preload was set to 30 mN. The attachment and retraction velocities were 5 µm s^−1^. Forces were deduced from the beam deflection under consideration of the spring constant [32]. The pull-off force was defined as the maximum tensile force obtained from the force-displacement curve. Based on the curvature radius of the probe and the indentation depth, the apparent contact area was estimated theoretically. The pull-off stress then was determined by dividing the pull-off force by the apparent contact area at preload [33]. The apparent contact area, A, varied with the indentation depth (i.e., the depth that the probe penetrates into the adhesive surface) and was calculated from the equation: ${A\;=\;\pi \;[R}^{2}-{\left(R-\delta \right)}^{2}]$, where R is the curvature radius of the glass probe, and δ is the indentation depth at preload. The normalized work of separation, W, was obtained by integrating the enclosed area of the stress-displacement curve in the tensile (adhesive) regime: $W=\int_{\Delta_{0}}^{\Delta_{1}}\sigma d\Delta$, where σ is the stress and Δ is the displacement (see Figure S1b in the Supplementary Material). Five positions on each sample were measured and the mean values were reported. 

The maximum shear stress of the dry adhesive film was determined by a displacement-controlled lap shear test in a tensile tester (Zwick Roell, Xforce P). Five specimens were prepared by attaching the dry adhesive film on a piece of glass plate, with an overlapping area of 8 cm² (width: 2 cm; length: 4 cm). Cross head moving speed was controlled at 5 mm min^−1^ until the film detached from the glass plate. The shear strength was calculated by dividing the maximum force by the overlapping area.

## 3. Results

### 3.1. Microstructure Results

Figure 1 gives an overview of the continuous roll-to-roll fabrication process. It illustrates the UV imprint unit (Figure 1a) and the surface structures on the micropatterned Ni shim (Figure 1b). Figure 1c displays the 10-cm wide adhesive films obtained from the process; it appeared opaque due to light scattering by the microstructures, although the material UA16 had a clear appearance. To facilitate the demolding of complex-shaped mushroom structures, which exhibited re-entrant features, the material selection for the template was very important. The properties of the two materials for the template (ePDMS) and the adhesive (UA16) are summarized in Table 1. The silicone-based (ePDMS) template had an elastic modulus of 0.76 MPa and a surprisingly high elongation at break of about 700%. The elastic modulus of UA16 was 359 MPa, which is relatively high compared to ePDMS, and an elongation at break of about 326%. Both materials exhibit high toughness, whereas the UA16 was stiff compared to the soft ePDMS. This combination provided excellent conditions for high structural integrity without the collapse of very fine structural features such as the mushroom tips and, in the same way, the ability to compensate for large deformations during demolding. However, the flexibility of the template meant that a certain degree of deformation could occur during the imprint process. These deformations were in turn reflected by variations in the morphology of the microstructures obtained upon demolding.

In Figure 2, scanning electron micrographs show the morphologies obtained for various compressive states of the template related to the distances between the template and the wet coating, d. For all d values between −13 and −515 μm, micropillars were obtained, but their morphology could be separated into two regimes: “no mushroom” and “with mushroom”. In addition, the group “with mushroom” exhibits micropillars with further distortions. The details are described below.

- “No mushroom” range (d in the range of −13 to −130 μm): At low compression of the template, micropillars without mushroom tip were formed (Figure 2a,b). Instead, the “no mushroom” pillars exhibited concave tip faces. The pillar heights were only 36 and 40 μm for d = −13 μm and −130 μm, respectively, which is below the original height of the cavities with a depth of 50 μm.
- “Mushroom” range (d in the range of −247 to −515 μm): With sufficient compression, microstructures with mushroom caps were generated (Figure 2c–h).

It should be noted that the shape of the pillars was slightly tapered, reflecting the structures in the Ni shim, which means that the pillar diameter at the connection to the backing layer was slightly smaller (45.9 µm) than below the mushroom tip (50 µm) (Table 2). The pillar diameter at the backing layer position remained similar for small compression (−13 μm > *d* > −305 μm) but was much smaller for d ≤ −363 μm. The angle between the tapered sidewall of the microstructure and the backing layer was designed to be slightly larger than 90° to reduce stress intensities at this location. This angle changed from above 90° to below 90° for d ≤ −363 μm (Table 2, Figure 2e–h). In addition to distortions of the microstructures, the backing layer deformed for d ≤ −468 μm (Figure 2g,h) associated with an increase of the pillar length to 59.3 μm and 58.3 μm for d = −468 μm and d = −515 μm, respectively. 

It is interesting that the required pressing distance is larger than the microcavity depth, which is very likely due to the flexibility of ePDMS template. As reported, additional pressure is necessary for imprinting to achieve better filling of liquid prepolymer in tiny cavities in mold; [34] however, in our experiment, it was impossible to measure the value of pressure applied on the flexible template. Instead, we use the “pressing distance” as a proxy for the degree of compression. This pressing distance will be highly dependent on the flexibility of the template. Flexible templates require larger pressing distance than rigid templates, in order to reach the same level of pressure. It means that the pressing distance is not equal to the prepolymer filling depth. In fact, the filling depth is restricted by the interaction between prepolymer and the template due to prepolymer viscosity. Also, it is unavoidable to lose some prepolymer by squeezing out, considering that the filling of resin with high viscosity into fine microcavities needs a certain compression.

### 3.2. Adhesion Results

The adhesion for the various microstructures was tested using the custom-built setup illustrated in Figure 3a. Apparent contact areas at the fixed preload of 30 mN and pull-off forces are depicted in Figure 3b. To correlate these results with the morphologies, the regimes “no mushroom” and “with mushroom” were highlighted. In addition, a color gradient reflects the degree of distortion within the “with mushroom” regime. The apparent contact area decreased with decreasing d until a minimum was reached at −363 μm. For d < −363 μm, the apparent contact area increased again. 

The trend in pull-off forces is in good agreement with the corresponding morphology: microstructures with “no mushroom” led to low adhesion, whereas “with mushroom” resulted in higher adhesion forces by up to a factor 5. In the mushroom regime, the pull-off force increased with decreasing d until a maximum of 28.3 mN was achieved at −363 μm. For higher compression, the pull-off force decreased again. The largest compression (d = −515 μm) was accompanied with a significant reduction of the pull-off force. Normalization of the forces by the calculated, apparent contact areas led to pull-off stress and work of separation values as shown in Figure 3c,d, respectively. Also, in terms of strength, the adhesion performance of microstructures without mushrooms was very low compared to the microstructures with mushrooms. In particular, microstructures generated with an intermediate compression (−247 > *d* > −422 μm) exhibited pull-off stresses larger than 100 kPa and work of separation larger than 1.4 J m^−2^. Maximum values were obtained for microstructures fabricated at d = −363 μm, where the pull-off stress and the work of separation were about 250 kPa and 2.5 J m^−2^, respectively. The outstanding adhesion performance is most likely related to the pronounced mushroom tip formation as well as the firm connection to the backing layer. In contrast, for d < −363 μm, the pull-off stress and work of separation decreased, revealing that excessive compression of the microstructures is detrimental to adhesion.

**Figure 4:**
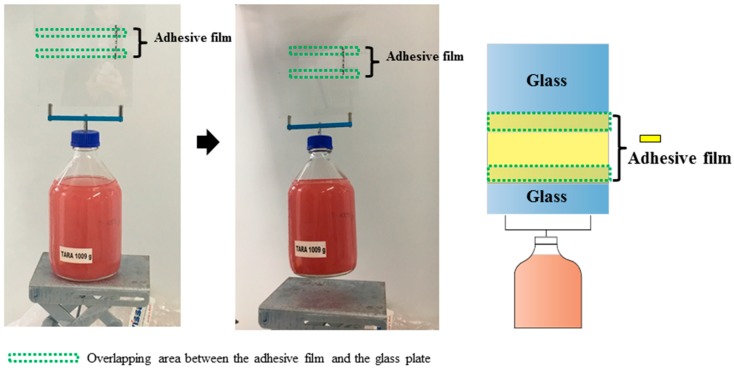
Demonstration of shear adhesion. Photograph showing a glass bottle (2000 ml, 1 kg) filled with 2 kg water mixed with red colorant (left); Illustration of the setup for the shear adhesion demonstration (right). The dry adhesive film was obtained at *d* = −363 µm. The overlap length was 10 cm; the total contact area was 14 cm².

To demonstrate shear adhesion, a water-filled flask with a total mass of 3 kg was fixated in shear with the dry adhesive film (generated with *d* = −363 µm) (Figure 4). Two pieces of glass were attached at each end of the adhesive film to eliminate possible bending and twisting. The overlapping areas between glass plates and adhesive film are marked with green squares in Figure 4. As only the upper overlapping region was exposed to gravitational forces, the contact area was 14 cm². A shear stress of 104.15 ± 36.24 kPa was obtained from tensile tests. Here, a glass plate and an adhesive film with an overlapping area of 8 cm² were oppositely loaded until detachment. 

## 4. Discussion 

The success of continuous microstructure fabrication by a roll-to-roll process was, first, due to suitable material selection for both the adhesive and the template. The aliphatic urethane diacrylate (UA16) exhibited a relatively high elastic modulus of 359 MPa, a high surface free energy of 40 mJ m^−2^ and, a moderate toughness with a large elongation at break about 326%. These properties led to high adhesion to smooth glass substrates, which in fact is higher than the typical adhesion of silicone-based microstructures [32,35,36]. The stiffness of the material also preserved the integrity of fine structural features such as the flaps of the mushroom tips when the structures were removed from the template. The viscosity of the wet coating was sufficiently low to fill the cavities during imprinting. The material cured within seconds by irradiation with UV light and was therefore suitable for a continuous process. To enable the demolding of the complex-shaped microstructures, outstanding flexibility of the template was essential [29]. The low elastic modulus (0.76 MPa) and the high toughness with an elongation of break of about 700% offered excellent conditions for that. The templates could be used without any further modifications due to their chemical inertness to the acrylate-based cross-linking reaction. The low-surface free energy (26 mJ m^−2^) greatly facilitated demolding [21].

We found that the flexibility of the template is decisive for the quality of the replicated microstructures, particularly, at high compression during fabrication [37]. The results obtained clearly demonstrate that the morphology of the micropillars could be modulated by adjusting the distance between the wet coating and the template, i.e., a variation of the “compression”. Sufficient compression was necessary to fabricate micropillars with mushroom tips (see Figure 2). This indicates that the cavities were not filled by capillary forces alone. The reason for that is most likely related to the combination of the surface free energy and viscosity of UA16 providing poor wetting on the ePDMS template. Nevertheless, cavities of the template could be filled by decreasing the distance between the template and wet coating during imprinting. We found that there exists an optimum where cavities were filled but not deformed. Too high pressures caused deformations of the template, which were in turn transferred into the shape of the microstructure. Consequently, microstructures were longer, and more importantly, the lower part of the microstructures was deformed (see Table 2, Figure 2f–h). Thus, the pillar diameter at the backing layer was thinner (smaller D) and the angle between the pillar sidewall and the backing layer transitioned from above to below 90°. 

The different morphologies of the microstructures resulted in different adhesion values, confirming that adhesion of micropatterned dry adhesives is quite sensitive to their geometrical design. In line with several previous studies [12,15,19,38], the samples without mushrooms were less adhesive than the mushroom-shaped microstructures (see Figure 3). The preload was kept constant in the adhesion tests, and therefore the apparent contact area varied depending on the stiffness of the samples. Higher stiffness of the sample resulted in smaller indentation depth of the spherical glass probe, which in turn led to smaller apparent contact area. This is a familiar complication of adhesion tests with spherical counter surfaces, which could be avoided only by tests with flat surfaces.

With decreasing distance between the template and the wet coating, the compression increased, which most probably led to thinner backing layers (backing layer thickness, *h*, see Table 2) and therefore stiffer samples with less apparent contact area. It is known that a thinner backing layer can result in a higher pull-off force because of a more efficient load sharing [8,39,40]. This trend was found for d ≥ −363 μm. For smaller d, the contact area increased further. As discussed above, microstructures fabricated under these conditions exhibited narrow necks close to the backing layer. This most likely leads to larger compliance, but in the same way, to a reduced resistance against buckling. Buckling of the microstructures typically lead to drastic reduction of adhesion [41,42], which most probably explains the reduced adhesion for d < −363 μm though the microstructures exhibited mushroom tips. The best adhesion performance was obtained for d = −363 μm. This optimum was associated with the thinnest backing layer without deteriorating its connection to the microstructures.

## 5. Conclusions

Process-material integration for a continuous roll-to-roll fabrication and excellent adhesion properties were realized by appropriate material selection, namely, UV-curable UA16 as material for the adhesive and ePDMS for the flexible template. The flexibility of the template allowed some elastic deformation, which varied as function of the distance between the template and the wet coating. The following conclusions can be drawn:

- Fabrication of mushroom-shaped microstructures exhibiting re-entrant features is possible by utilization of flexible templates.
- Compression between the template and the wet coating is required to fill the cavities of the silicone-based mold. Insufficient compression leads to microstructures without mushrooms accompanied by low-adhesion performance.
- The compression could be controlled by the distance between the flexible template and the wet coating. Variations of the distance result in variations of the morphology, which in turn control the adhesion performance. Best adhesion results were obtained for microstructures generated using the shortest distance before they were distorted due to template deformation.
- In addition to normal adhesion, the micropatterned dry adhesive film exhibited remarkable shear adhesion, demonstrating its high potential for applications where normal and shear adhesion are required.

## Figures and Tables

**Table 1:** Material properties of UA16 (adhesive material) and ePDMS (template).

Material	Description	Young’s Modulus (MPa)	Elongation at Break (%)	Surface Free Energy (mJ m^−2^)	Viscosity at 25 °C (Pa s)
UA16	Aliphatic urethane diacrylate oligomer	359	326	40.26	13.7
ePDMS	Elastosil M4601	0.76	700	25.82	--

**Table 2:** Microstructure parameters: Angle between the micropillar side wall and the backing layer, θ, and pillar diameter at connection to the backing layer, D, at varied distances between the template and the wet coating, d. Backing layer thickness, *h*.

d (µm)	D (µm)	θ (°)	*h* (µm)
−13	45.7	>90	45.7
−130	44.7	>90	41.2
−247	45.4	>90	37.5
−305	45.1	>90	34.6
−363	40.8	<90	31.2
−422	37.7	<90	28.6
−468	32.7	<90	19.6
−515	32.4	<90	15.8
On Ni shim	45.9	>90	-

## Supplementary Materials

The following are available online at http://www.mdpi.com/1996-1944/12/1/97/s1. Figure S1: (a) Illustration of adhesion test device. (b) Force-displacement curve from a normal adhesion test with a spherical glass probe; Table S1: Surface free energy of UA16 and ePDMS as determined by contact angle (CA) measurements.
