# Supplementary material for: Roll-to-Roll Manufacturing of Micropatterned Adhesives by Template Compression
Source: Materials (Basel). 2018 Dec 29;12(1):97. doi: 10.3390/ma12010097 (PMC6337371; doi:10.3390/ma12010097)
Supplement: Supplementary file 1 [file materials-12-00097-s001.pdf]

Table S1. Surface free energy of UA16 and ePDMS as determined by contact angle (CA) measurements.

	CA $_{water}$ (°)	CA $_{n\text{-}hexadecane}$ (°)	Dispersive surface free energy (mJ m^{-2})	Polar surface free energy (mJ m^{-2})	Total surface free energy (mJ m^{-2})
UA16	75.97	3.59	27.42	12.84	40.26
ePDMS	105.77	29.13	24.20	1.62	25.82

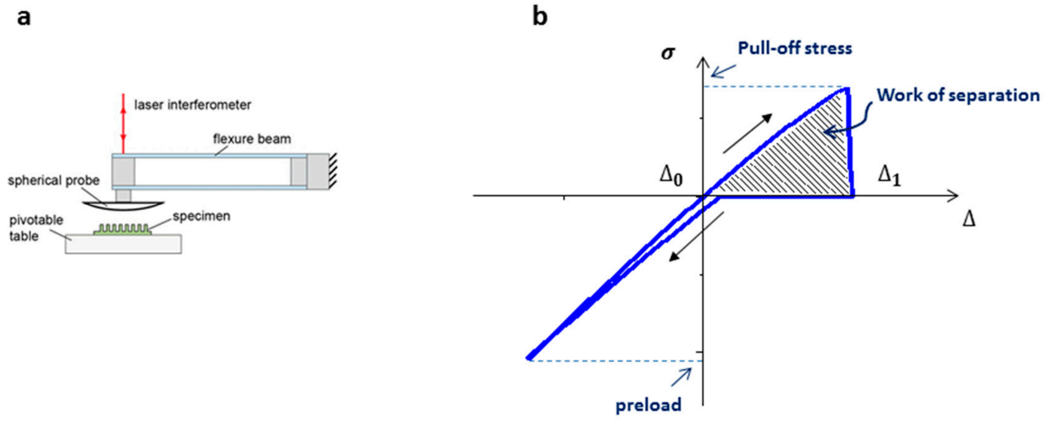

Figure S1. a) Illustration of adhesion test device. **b)** Force-displacement curve from a normal adhesion test with a spherical glass probe.
